# Supplementary material for: Prevalence of SARS-CoV-2 in an area of unrestricted viral circulation: Mass seroepidemiological screening in Castiglione d’Adda, Italy
Source: PLoS One. 2021 Feb 24;16(2):e0246513. doi: 10.1371/journal.pone.0246513 (PMC7904134; doi:10.1371/journal.pone.0246513)
Supplement: S1 Table — Numerical variables (namely: age and BMI) are presented as means and standard deviations. Categorical variables are presented as total counts and percentages. BMI was calculated for 3668 subjects aged ≥20 years. RCF: Residential Care Facilities; BMI: Body Mass Index; CAD: Coronary Artery Disease; MI: Miocardial Infarction; COPD: Chronic Obstructive Pulmonary Disease. (DOCX) [file pone.0246513.s001.docx]

**S1 Table: Characteristics of 509 subjects in the random sample**

Numerical variables (namely: age and BMI) are presented as means and standard deviations. Categorical variables are presented as total counts and percentages. BMI was calculated for 3668 subjects aged ≥20 years. RCF: Residential Care Facilities; BMI: Body Mass Index; CAD: Coronary Artery Disease; MI: Miocardial Infarction; COPD: Chronic Obstructive Pulmonary Disease;

|  | **IgG negative**  **(n=394)** | **IgG positive**  **(n=115)** |
| --- | --- | --- |
| Gender (Female) | 200 (50·8%) | 49 (42·6%) |
| Age (years) | 46·0, 20·6 | 55·4, 19·5 |
| Contact with verified case | 93 (23·6%) | 61 (53·0%) |
| Smoker | 92 (23·4%) | 10 (8·7%) |
| Cardiovascular diseases  - CAD/MI  - Arrhythmias  - Hypertension  - Other  At least one of the above: | 10 (2·5%)  14 (3·6%)  68 (17·3%)  14 (3·6%)  84 (21·3%) | 3 (4·3%)  5 (4·3%)  32 (27·8%)  14 (12·2%)  47 (40·9%) |
| Rheumatic diseases | 19 (4·8%) | 11 (9·6%) |
| Diabetes mellitus | 12 (3·0%) | 6 (6·2%) |
| Chronic Lung diseases  - Asthma  - COPD  - Other  At least one of the above: | 20 (5·1%)  1 (0·3%)  9 (2·3%)  29 (7·4%) | 2 (1·7%)  1 (0·9%)  4 (3·5%)  7 (6·1%) |
| Oncological pathologies  Solid Tumors  Oncochematological  At least one of the above: | 20 (5·1%)  2 (0·5%)  22 (5·6%) | 6 (5·2%)  2 (1·7%)  8 (7·0%) |
| Symptomatic  - Fever  - Cough  - Anosmia  - Dysgeusia  - Dyspnea  - Rush:  - Arthromyalgia  At least one of the above:  Other symptoms | 65 (16·5%)  57 (14·5%)  23 (5·8%)  27 (6·9%)  23 (5·8%)  11 (2·8%)  34 (8·6%)  124 (31·5%)  54 (13·7%) | 66 (57·4%)  31 (27·0%)  37 (32·2%)  46 (40·0%)  13 (11·3%)  4 (3·5%)  36 (31·3%)  89 (77·4%)  23 (20·0%) |
