# Supplementary material for: Prevalence of SARS-CoV-2 in an area of unrestricted viral circulation: Mass seroepidemiological screening in Castiglione d’Adda, Italy
Source: PLoS One. 2021 Feb 24;16(2):e0246513. doi: 10.1371/journal.pone.0246513 (PMC7904134; doi:10.1371/journal.pone.0246513)
Supplement: S2 Table — Initial model: multivariate model including all the considered factors included as independent variables. Final model: Model selected by the backward selection procedure. Linear term and non-linear term refer to the regression model coefficients required for the estimation of the non-linear effect of age. OR: Odd Ratio, Est: estimate, C.I.: Confidence interval. BMI: Body Mass Index; CAD: Coronary Artery Disease; MI: Myocardial Infarction; COPD: Chronic Obstructive Pulmonary Disease. (DOCX) [file pone.0246513.s002.docx]

**S2 Table: Association between positivity to IgG for CLIAs and the characteristics of interest**

Initial model: multivariate model including all the considered factors included as independent variables. Final model: Model selected by the backward selection procedure. Linear term and non-linear term refer to the regression model coefficients required for the estimation of the non-linear effect of age. OR: Odd Ratio, Est: estimate, C.I.: Confidence interval. BMI: Body Mass Index; CAD: Coronary Artery Disease; MI: Myocardial Infarction; COPD: Chronic Obstructive Pulmonary Disease;

|  | **Unadjusted OR** | | **Adjusted OR** | |
| --- | --- | --- | --- | --- |
|  | **Est (95% C.I.)** | **p-value** | **Est (95% C.I.)** | **p-value** |
| Gender: Female | 1·39 (0·76, 2·53) | 0·2824 | 1·00 (0·81, 1·24) | 0·9731 |
| Age (years) | 1·02 (1·02, 1·03) | <0·0001 | 1·03 (1·03, 1·04) | <0·0001 |
| Contact with verified case: | 3·66 (3·17, 4·21) | <0·0001 | 3·73 (2·57, 5·43) | <0·0001 |
| Smoker: | 0·31 (0·20, 0·48) | <0·0001 | 0·34 (0·15, 0·78) | 0·0110 |
| Cardiovascular diseases  - Hypertension:  - CAD/arr/MI/Other: | 1·85 (1·14, 3·01)  2·71 (1·61, 4·57) | 0,0134  0·0002 |  |  |
| Rheumatic diseases: | 2·09 (1·12, 3·89) | 0,0203 |  |  |
| Diabetes mellitus: | 1·75 (0·92, 3·33) | 0,0871 |  |  |
| Chronic Lung diseases  At least one: | 0·82 (0·38, 1·76) | 0·6030 |  |  |
| Oncological pathologies  At least one: | 1·26 (0·89, 1·79) | 0,1841 |  |  |
| Fever:  Cough:  Anosmia:  Dysgeusia:  Dyspnea:  Rush:  Arthromyalgia:  Other symptoms: | 6·82 (4·33, 10·74)  2·18 (1·51, 3·16)  7·65 (5·40, 10·84)  9·06 (6·68, 12·29)  2·06 (1·00, 4·22)  1·25 (0·38, 4·17)  4·83 (3·09, 7·54)  1·57 (0·92, 2·68) | <0·0001  <0·0001  <0·0001  <0·0001  0·0492  0·7109  <0·0001  0·0957 | 3·86 (2·04, 7,31)  7·05 (3·08, 16·16)  0·39 (0·16, 0·99) | <0·0001  <0·0001  0·0493 |
