# Supplementary material for: Prevalence of SARS-CoV-2 in an area of unrestricted viral circulation: Mass seroepidemiological screening in Castiglione d’Adda, Italy
Source: PLoS One. 2021 Feb 24;16(2):e0246513. doi: 10.1371/journal.pone.0246513 (PMC7904134; doi:10.1371/journal.pone.0246513)
Supplement: S1 Appendix — (DOCX) [file pone.0246513.s003.docx]

**S1 Appendix: Administered epidemiological and clinical questionnaire**

**1. GENERAL INFORMATION**

Name: ________________________________Surname: _________________________________

Sex: M F Date of birth: (DD|MM|YYYY): |__|__|__|__|__|__|__|__|

Resident in Castiglione d’Adda: YES NO

Domiciled in Castiglione d’Adda: YES NO

Employed in Castiglione d’Adda: YES NO

If not, specify workplace: ___________________________________________________________

Occupation: ____________________________________________________________________

Locations attended (even if outside Castiglione d’Adda) in the first months of 2020:

Bar/Pub |__| Nightclub |__| Social Club |__| Sports center |__|

Specify name and location: _________________________________________________________

Smoker YES NO since (year) |__|__|__|__| Sigarettes/day |__|__|

Have you ever been vaccinated against influenza (the flu)? YES NO

If so, was it during the past flu season (2019/2020)?: YES NO

Have you ever had contact with confirmed COVID-19 cases (swab-positive or hospitalized for COVID-19)? YES NO

If so:

Who (Name)? _______________________

When (date of last contact)? ___________________

Have you ever had a swab taken for SARS-CoV-2? YES NO

If so:

When (date)? ________________

What was the result? (select result) POS NEG I DON’T KNOW/DON’T REMEMBER

If POSITIVE, were you hospitalized? YES NO

If so, were you in the ICU? YES NO

**2. DO YOU CURRENTLY SUFFER FROM OR HAVE YOU EVER SUFFERED FROM (MARK WITH X IF RELEVANT):**

- CARDIOVASCULAR DISEASE
  - HYPERTENSION (HIGH BLOOD PRESSURE)
  - MYOCARDIAL INFARCTION (HEART ATTACK) OR OTHER CORONARY DISEASE (ANGINA PECTORIS)
  - ARRHYTHMIAS
  - OTHER (please specify): _____________________________________
- RHEUMATIC/AUTOIMMUNE DISEASE
  - PLEASE SPECIFY: _______________________________________
- DIABETES MELLITUS
- CHRONIC LUNG DISEASE
  - ASTHMA
  - COPD (CHRONIC BRONCHITIS/EMPHYSEMA)
  - OTHER (please specify): ______________________________________
- ONCOLOGIC DISEASE (CANCER/TUMORS)
  - SOLID TUMORS
  - ONCOHEMATIC DISEASE (LEUKEMIA/LYMPHOMA)

**3. CLINICAL INFORMATION**

WEIGHT (KG): |__|__|__| HEIGHT (CM): |__|__|__|

**SINCE 01/02/2020 TO TODAY, HAVE YOU EVER SUFFERED FROM:**

- FEVER
  - MAXIMUM AXILLARY TEMPERATURE (in °C): |__|__|.|__|
  - START DATE: __________________ END DATE: __________________
  - SYMPTOM PRESENT IN THE PAST 14 DAYS
- COUGH
  - START DATE: __________________ END DATE: __________________
  - SYMPTOM PRESENT IN THE PAST 14 DAYS
- REDUCED OR ALTERED SENSE OF SMELL OR TASTE
  - START DATE: __________________ END DATE: __________________
  - SYMPTOM PRESENT IN THE PAST 14 DAYS
- DIFFICULTY BREATHING
  - START DATE: __________________ END DATE: __________________
  - SYMPTOM PRESENT IN THE PAST 14 DAYS
- MUSCLE OR BONE ACHES AND PAINS
  - START DATE: __________________ END DATE: __________________
  - SYMPTOM PRESENT IN THE PAST 14 DAYS
- SKIN RASH
  - START DATE: __________________ END DATE: __________________
  - SYMPTOM PRESENT IN THE PAST 14 DAYS
- OTHER (please specify): ______________________________________________________
  - START DATE: __________________ END DATE: __________________
  - SYMPTOM PRESENT IN THE PAST 14 DAYS
