# Supplementary material for: Prevalence of SARS-CoV-2 in an area of unrestricted viral circulation: Mass seroepidemiological screening in Castiglione d’Adda, Italy
Source: PLoS One. 2021 Feb 24;16(2):e0246513. doi: 10.1371/journal.pone.0246513 (PMC7904134; doi:10.1371/journal.pone.0246513)
Supplement: Attachment — Submitted filename: response rev.doc [file pone.0246513.s005.doc]

Milan, 25^th^ of August 2020

To the Editor-in-Chief,

PLoS ONE

We thank you for the interest shown in our work and your careful revision.

Editorial and reviewer’s comments with our responses (italic) are reported below.

Kindest regards

Gabriele Pagani MD

1. Please ensure that your manuscript meets PLOS ONE's style requirements, including those for file naming. The PLOS ONE style templates can be found at

<https://journals.plos.org/plosone/s/file?id=wjVg/PLOSOne_formatting_sample_main_body.pdf> and

<https://journals.plos.org/plosone/s/file?id=ba62/PLOSOne_formatting_sample_title_authors_affiliations.pdf>

2. Thank you for including your ethics statement:  "This study was approved by University of Milan's Ethical Committee (allegato 1 Comitato Etico 21.04.20 - parere numero 35/20).".

Please provide additional details regarding participant consent. In the ethics statement in the Methods and online submission information, please ensure that you have specified (1) whether consent was informed and (2) what type you obtained (for instance, written or verbal, and if verbal, how it was documented and witnessed). If your study included minors, state whether you obtained consent from parents or guardians. If the need for consent was waived by the ethics committee, please include this information.

If you are reporting a retrospective study of medical records or archived samples, please ensure that you have discussed whether all data were fully anonymized before you accessed them and/or whether the IRB or ethics committee waived the requirement for informed consent. If patients provided informed written consent to have data from their medical records used in research, please include this information.

Once you have amended this/these statement(s) in the Methods section of the manuscript, please add the same text to the “Ethics Statement” field of the submission form (via “Edit Submission”).

For additional information about PLOS ONE ethical requirements for human subjects research, please refer to [http://journals.plos.org/plosone/s/submission-guidelines#loc-human-subjects-research](http://journals.plos.org/plosone/s/submission-guidelines#loc-human-subjects-research).

*Ethics statement has been amended as per journal’s requirements and moved to the “methods” section.*

3. Thank you for stating the following in the Financial Disclosure section:

"This study was realized thanks to non-conditioning financial contributions from: CISOM (Corpo Italiano di Soccorso dell'Ordine di Malta, [https://www.cisom.org](https://www.cisom.org/)), FC Internazionale Milano (<https://www.inter.it/>), SFD s.p.a/Fondazione SAME (<https://fondazionesame.it/>), Emporio Armani Olimpia Milano (<http://www.olimpiamilano.com/>) through donations to the Dipartimento di Scienze Biomediche e Cliniche (DIBIC) of the University of Milan and to Banca Mediolanum (<https://www.bancamediolanum.it/>) through donation to ASST Fatebenefratelli-Sacco, Milano.
Mylan Italia s.p.a. donated the rapid tests.
The donations were used to cover the expenses related to personal protective equipments, materials, laboratory processing and personnel costs.
The funders had no role in study design, data collection and analysis, decision to publish, or preparation of the manuscript."

We note that you received funding from a commercial source: , FC Internazionale Milano, Emporio Armani Olimpia Milano

Please provide an amended Competing Interests Statement that explicitly states this commercial funder, along with any other [relevant declarations](about:blank) relating to employment, consultancy, patents, products in development, marketed products, etc.

Within this Competing Interests Statement, please confirm that this does not alter your adherence to all PLOS ONE policies on sharing data and materials by including the following statement: "This does not alter our adherence to PLOS ONE policies on sharing data and materials.” (as detailed online in our guide for authors [http://journals.plos.org/plosone/s/competing-interests](about:blank)).  If there are restrictions on sharing of data and/or materials, please state these. Please note that we cannot proceed with consideration of your article until this information has been declared.

Please include your amended Competing Interests Statement within your cover letter. We will change the online submission form on your behalf.

4. Thank you for stating the following in the Competing Interests section:

"The authors have declared that no competing interests exist."

We note that one or more of the authors are employed by a commercial company: Medispa s.r.l., Synlab Italia s.p.a..

4.1. Please provide an amended Funding Statement declaring this commercial affiliation, as well as a statement regarding the Role of Funders in your study. If the funding organization did not play a role in the study design, data collection and analysis, decision to publish, or preparation of the manuscript and only provided financial support in the form of authors' salaries and/or research materials, please review your statements relating to the author contributions, and ensure you have specifically and accurately indicated the role(s) that these authors had in your study. You can update author roles in the Author Contributions section of the online submission form.

Please also include the following statement within your amended Funding Statement.

“The funder provided support in the form of salaries for authors [insert relevant initials], but did not have any additional role in the study design, data collection and analysis, decision to publish, or preparation of the manuscript. The specific roles of these authors are articulated in the ‘author contributions’ section.”

If your commercial affiliation did play a role in your study, please state and explain this role within your updated Funding Statement.

4.2. Please also provide an updated Competing Interests Statement declaring this commercial affiliation along with any other relevant declarations relating to employment, consultancy, patents, products in development, or marketed products, etc.

Within your Competing Interests Statement, please confirm that this commercial affiliation does not alter your adherence to all PLOS ONE policies on sharing data and materials by including the following statement: "This does not alter our adherence to  PLOS ONE policies on sharing data and materials.” (as detailed online in our guide for authors [http://journals.plos.org/plosone/s/competing-interests](about:blank)) . If this adherence statement is not accurate and  there are restrictions on sharing of data and/or materials, please state these. Please note that we cannot proceed with consideration of your article until this information has been declared.

Please include both an updated Funding Statement and Competing Interests Statement in your cover letter. We will change the online submission form on your behalf.

Please know it is PLOS ONE policy for corresponding authors to declare, on behalf of all authors, all potential competing interests for the purposes of transparency. PLOS defines a competing interest as anything that interferes with, or could reasonably be perceived as interfering with, the full and objective presentation, peer review, editorial decision-making, or publication of research or non-research articles submitted to one of the journals. Competing interests can be financial or non-financial, professional, or personal. Competing interests can arise in relationship to an organization or another person. Please follow this link to our website for more details on competing interests: [http://journals.plos.org/plosone/s/competing-interests](about:blank)

*Funding Statement has been amended as per journal’s requirements. The corrected version is reported at the end of this letter.*

5. Your ethics statement should only appear in the Methods section of your manuscript. If your ethics statement is written in any section besides the Methods, please move it to the Methods section and delete it from any other section. Please ensure that your ethics statement is included in your manuscript, as the ethics statement entered into the online submission form will not be published alongside your manuscript.

*Ethics statement has been moved to the right section.*

6. Please include captions for your Supporting Information files at the end of your manuscript, and update any in-text citations to match accordingly. Please see our Supporting Information guidelines for more information: [http://journals.plos.org/plosone/s/supporting-information](about:blank).

*Supporting Tables have been amended*

**Comments to the Author**

Reviewer #1: Minor issues
Write up is pretty easy read and understandable, however, please note some Syntax issues
For examples:
Vocabulary _ A different word could be used instead of diffusion that might make more sense in this context
Long Sentences_ 3rd paragraph; “Recently, lateral…..
Spelling _ “seferely” should be severely.

*The authors thank you for your suggestions, syntax errors pointed out by reviewer were corrected.*

Technical:
Please explain why 2 different tests to run NPSs and note the differences between the two, including their sensitivity/specificity ratings.

*Due to the dramatic escalation of the epidemic in Northern Italy, not all PCR reagents were readily available. We added this explanation to the methods section and included technical specifications of both tests in the references.*

Missing several necessary elements, including information on sample storage (temperature, location, etc.), average time intervals between sample collection and processing, and sample destruction procedures after processing was complete.

*Altough many of the required information were already stated in the protocol (published on a pre-print server and referenced in the methods section) we added them to the text for clarity.*

Were samples given a unique identifier for blinding the study and perhaps privacy?

*Samples were identified by a standard label (which included a barcode, name, surname and date of birth). Authors did not consider necessary to blind samples.
Moreover, a nominal medical report of NPSs’ results was required by Italian law, as it was necessary to file a communicable disease’s report to the health authorities.*

The reason for not measure the BMI of people under the age of 19 – This may got to Discussion.

*As per WHO directions (referenced in text), BMI calculation is applicable only for subjects over 19 years old (while the body mass for subjects younger than 19 should be standardized using centiles).*

Results:
I had to dig through the paper to find out how many people were tested for the different tests/kits. For example, on page 10, last paragraph, the authors state that 1330 people were tested using the NPS method. On page 11, under Diagnostic accuracy, the authors note that 562 subjects were selected for the CLIA serological tests and PCR.
Also, please explain well if one person might have been randomly selected for more than one test. This should be addressed in the methods section as well.

*As explained in the methods section, 562 subjects were randomized to be tested by NPS and serologies on venous blood regardless of the results of the RICT, while everyone with a positive RICT would be tested with both serologies and NPS (excluding those with a documented history of a positive NPS followed by two negatives, as per Italian laws at the time). Consequently, 1330 represents the total number of NPS, including those tested because they were part of the random sample and those with a positive RICT. It is important to note that the two groups may overlap, as a random subject could test positive to RICT.
This has been added to the text in the methods section, for clarity.*

Tables design- Gender (Female) issue is confusing; how about the male data?

*Tables have been amended as per reviewer request.*

“The strong association observed between” should include statistical data for clarity.

*Adjusted ORs were included in the text.*

**Funding Statement**

This study was realized thanks to non-conditioning financial contributions from: CISOM (Corpo Italiano di Soccorso dell'Ordine di Malta), FC Internazionale Milano, SFD s.p.a, Emporio Armani Olimpia Milano through donations to the Dipartimento di Scienze Biomediche e Cliniche (DIBIC) of the University of Milan; from Banca Mediolanum through donation to ASST Fatebenefratelli-Sacco, Milano.

Mylan Italia s.p.a. donated the rapid tests.

The donations were used to cover the expenses related to personal protective equipments, materials, laboratory processing and personnel costs.

None of the funding sources were involved in data collection, analysis or intepretation; trial design; patient recruitment; or any aspect pertinent to the study.

In particular none of the commercial funders (FC Internazionale Milano, Emporio Armani Olimpia Milano, SFD s.p.a. and Banca Mediolanum s.p.a.) had any role in data collection, analysis or intepretation; trial design; patient recruitment; or any aspect pertinent to the study, nor they have any commercial interest (e.g. consultancy, patents, products in development, marketed products, etc). This does not alter our adherence to PLOS ONE policies on sharing data and materials.

Medispa s.r.l. provided support in the form of salaries for authors RR and AP; Synlab s.p.a. provided support in the form of salaries for author CO; both funders did not have any additional role in the study design, data collection and analysis, decision to publish, or preparation of the manuscript. The specific roles of these authors are articulated in the ‘author contributions’ section.
